# Effect of high‐frequency repetitive transcranial magnetic stimulation over M1 for consciousness recovery after traumatic brain injury

**DOI:** 10.1002/brb3.2971

**Published:** 2023-03-28

**Authors:** Longbin Shen, Yixuan Huang, Yujun Liao, Xiaona Yin, Yulin Huang, Jianlin Ou, Hui Ouyang, Zhuoming Chen, Jinyi Long

**Affiliations:** ^1^ Department of Rehabilitation Medicine The First Affiliated Hospital of Jinan University Guangzhou Guangdong China; ^2^ Graduate School Gimcheon University Gimcheon South Korea; ^3^ Department of Rehabilitation Medicine The Fifth Affiliated Hospital of Guangzhou Medical University Guangzhou Guangdong China; ^4^ Department of Rehabilitation Shenzhen Longhua Maternity & Child Healthcare Hospital Shenzhen Guangdong China; ^5^ College of Information Science and Technology Jinan University Guangzhou Guangdong China

**Keywords:** disorders of consciousness, M1, neuroregulation, rTMS

## Abstract

**Background:**

The brain area stimulated during repetitive transcranial magnetic stimulation (rTMS) treatment is important in altered states of consciousness. However, the functional contribution of the M1 region during the treatment of high‐frequency rTMS remains unclear.

**Objective:**

The aim of this study was to examine the clinical [the Glasgow coma scale (GCS) and the coma recovery scale‐revised (CRS‐R)] and neurophysiological (EEG reactivity and SSEP) responses in vegetative state (VS) patients following traumatic brain injury (TBI) before and after a protocol of high‐frequency rTMS over the M1 region.

**Methods:**

Ninety‐nine patients in a VS following TBI were recruited so that their clinical and neurophysiological responses could be evaluated in this study. These patients were randomly allocated into three experimental groups: rTMS over the M1 region (test group; *n* = 33), rTMS over the left dorsolateral prefrontal cortex (DLPFC) (control group; *n* = 33) and placebo rTMS over the M1 region (placebo group; *n* = 33). Each rTMS treatment lasted 20 min and was carried out once a day. The duration of this protocol was a month with 20 treatments (5 times per week) occurring with that time.

**Results:**

We found that the clinical and neurophysiological responses improved after treatment in the test, control, and placebo groups; the improvement was highest in the test group compared to that in the control and placebo groups.

**Conclusions:**

Our results demonstrate an effective method of high‐frequency rTMS over the M1 region for consciousness recovery after severe brain injury.

## Graphical abstract

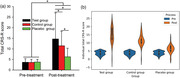

## 1 INTRODUCTION

Traumatic brain injury (TBI) is an injury caused by external violence directly or indirectly acting on the head. This is currently the primary factor leading to death and disability among young people internationally (Giacino & Trott, 2004). With economic development and the aging of the population, an increase in automobile traffic accidents, the incidence of TBI is increasing yearly (Abelson‐Mitchell, 2008; Maas et al., 2008). TBI is recognized as the serious world health problem that has not yet been resolved internationally. Treatment to help patients with craniocerebral injury recover their consciousness and return to society has received increasing attention (Li & Jiang, 2012; Sveen et al., 2022). However, the current research and treatment guidelines for TBI are basically focused on the rescue management of critically ill patients. For example, in the early stage, the goal was to reduce mortality (Iaccarino et al., 2015; Potapov et al., 2016). Now, consciousness disorder has become one of the most difficult problems in rehabilitation and awakening after TBI (Knight et al., 2005; Potapov et al., 2016).After the occurrence of severe TBI, most patients will generally be in a persistent vegetative state (VS) (Kondziella et al., 2020). A VS refers to a severely impaired state of consciousness after brain injury, and in which the presence of consciousness has not been detected clinically. The normal cycle of arousal is usually accompanied by a half‐asleep and half‐awake state that is close to the normal cycle of consciousness. The functions of the brainstem and thalamus are relatively preserved, but the functional connection of the cerebral cortex is generally damaged or missing (Manganotti et al., 2013). Electroencephalogram (EEG) activity is generally moderately or severely abnormal. The somatosensory evoked potentials on the upper limb are mainly prolonged to abnormal central conduction time (CCT) on one or both sides (Seel et al., 2010). It is generally believed that the development of a VS is divided into two stages. One is the worse permanent vegetative state stage, and the other is the better MCS stage. Many studies suggest that Patients in the MCS stage will have a greater chance of regaining consciousness (Giacino & Whyte, 2005; Lombardi et al., 2002; Manganotti et al., 2013). Therefore, clinically returning VS patients to an MCS state is of great help in promoting wakefulness.

Using neurostimulation technology for the treatment of severe brain injury to restore cognitive and physiological functions is considered to be an innovative and promising clinical treatment approach (Manganotti et al., 2013). Deep brain stimulation (DBS) experimental studies have proposed that DBS intervention may reestablish connections and maintain neuronal activity in the forebrain neurons that are disconnected; therefore, this approach could be used to treat Parkinson's disease, depression, etc. However, DBS is an invasive operation; there is a certain risk of surgery and complications, and clinical trials have not yet been conducted (Glannon, 2008; Schiff, 2012). Therefore, the clinical use of DBS is still very limited and cannot truly benefit VS patients. The current clinical noninvasive stimulation techniques mainly include single pulse transcranial magnetic stimulation (TMS) and repetitive transcranial magnetic stimulation (rTMS). Single pulse TMS has been shown to be able to effectively assess the excitability and conduction integrity of the motor area of the cerebral cortex, whereas rTMS has been shown to be able to induce changes in cerebral cortex function under normal and brain damage. rTMS is a noninvasive neuroelectro physiological treatment technique established on the basis of TMS in 1992 (Rickels et al., 2010). It can detect and regulate the activity of the cerebral cortex and has achieved satisfactory effects in the treatment of epilepsy, Parkinson's disease, cognitive dysfunction, psychosis and depression (George, 2010; Kodama et al., 2011; Sun et al., 2011). The working principle of rTMS is the induction of an electric field through a time‐varying magnetic field, causing secondary currents in adjacent nerve tissues (Barker et al., 1985). It activates the cortex by inducing an electric current, thereby changing the physiological processes in the brain. At the same time, by changing the excitability of the cerebral cortex and changing the cortical metabolism and cerebral blood flow, it affects the neurotransmitters and their transmission in the brain, increases the reversibility of damaged cells, and promotes the recovery of brain function (Hosono et al., 2008; Kito et al., 2008). When using rTMS, there are many factors would affect patients' awakening, such as the frequency of rTMS stimulation and the location of stimulation (He et al., 2018; Pink et al., 2021; Rossi et al., 2009; Xia et al., 2017).

In our recent study, it was found that using high‐frequency rTMS to stimulate the injured part of the patient's affected side has a positive effect on the recovery of consciousness after TBI (Shen et al., 2019). In patients with effective treatment, it was found that the “motor” score in the CRS‐R scale improved significantly, and the patient's prognosis was also better. Therefore, we propose that replacing the stimulation site with the M1 region may be more helpful in recovering patient consciousness. Therefore, if the clinical sample size can be further increased, it would be of great clinical significance to clarify the effective stimulation location of rTMS on recovering from a VS through randomized controlled studies.

In summary, this study intends to use 20 Hz high‐frequency rTMS to stimulate the M1 region in VS patients with a consciousness disorder for 1–3 months after TBI. A control experiment with the traditional stimulation of the damaged part of the affected side of the brain was performed to evaluate whether patients can enter an MCS from a VS more effectively after treatment, thereby optimizing clinical treatment methods and increasing the rate of wakefulness in patients with TBI.

## 2 METHODS

### 2.1 Patients

From September 2017 to September 2019, 105 VS patients with a consciousness disorder were recruited for this study from the Department of Neurosurgery, Intensive Care Unit (ICU), and Rehabilitation Department of the First Affiliated Hospital of Jinan University. According to the random number table, they were divided into a test group, control group and placebo group at a ratio of 1:1:1 in a sealed envelope. A total of 6 patients withdrew due to personal reasons and were unable to continue hospitalization (drop‐out rate 5.7%). A total of 99 patients completed the trial: 33 in the experimental group, 33 in the control group, and 33 in the placebo group. The experiment adopted a double‐blind method; that is, the randomized patients or their family members were unaware of whether they were in the experimental group or the control group, and the rTMS operator was also unaware of the trial design.

The inclusion criteria were as follows: (1) all patients met the diagnostic criteria for TBI and cognitive dysfunction based on the Chinese Guidelines for Diagnosis and Treatment of Vascular Cognitive Impairment (Wang, 2017); (2) all patients were diagnosed by MRI imaging examination, the course of disease was 1–3 months and were aged 20–80 years, if the course of VS patients is less than 1 month, their conditions are relatively unstable at the moment, and there is a certain risk of epilepsy, which could not be suitable for rehabilitation intervention. When VS patients have a course of more than 3 months, they may miss the optimal rehabilitation intervention period and the patients'prognosis would be poor; (3) all patients were in stable condition, had no or very low risk of epilepsy, and were in the VS/UWS stage of consciousness disorder as determined by GCS and CRS‐R assessments; (4) the family members voluntarily signed informed consent without formal rehabilitation training for consciousness disorder before the intervention.

The exclusion criteria were as follows: (1) individuals were critically ill and had unstable vital signs; (2) individuals had intracranial indwelling metal objects, cranial bone flaps, and skull defects; (3) patients who had a history of head injury or cardiovascular and cerebrovascular diseases.

### 2.2 Treatment

The experimental group, the control group and the placebo group all used post‐TBI secondary prevention medication. Except for the different methods of rTMS treatment, the three groups, including medication, conventional rehabilitation treatment and treatment prescriptions, were all consistent. Among them, conventional rehabilitation treatment included median nerve electrical stimulation, passive motor of limbs, and hyperbaric oxygen therapy. The three groups of treatment prescriptions were rTMS treatment for 20 min; conventional rehabilitation therapy for 20 min of median nerve electrical stimulation; 30 min of passive movement of limbs; and 40 min of hyperbaric oxygen treatment. The total time needed for all three treatment groups was 110 min and patients were treated once a day, 5 days a week; the course of treatment was 4 weeks.

The treatment method of the experimental group: the patients received conventional rehabilitation treatment and high‐frequency rTMS treatment with a frequency of 20 Hz over the M1 of the affected hemisphere.

The treatment method of the control group: the patients received conventional rehabilitation therapy and conventional rTMS treatment with a frequency of 20 Hz over the left dorsolateral prefrontal cortex (DLPFC).

The procedure for the placebo group: subjects in the placebo group received conventional rehabilitation treatment and fake rTMS stimulation (the stimulation parameters were the same as those of the test group).

### 2.3 Stimulation procedures

rTMS was performed using a CCY‐1 stimulator (Wuhan Yiruide Company Ltd, CH). TMS was delivered through a figure‐eight focal coil with a posterior–anterior (PA, coil handle 45° to the midline) coil orientation. Each patient underwent a session of 8000 stimuli delivered in 400 trains of 20 Hz rTMS at 90% of resting motor threshold (RMT). Each train lasted 1 s with a 2 s inter train pause. The total time of intervention was 20 min.

Before treatment, the patient was in a lying position, and the surface EMG electrode was placed on the abductor pollicis brevis (APB) on the affected side using Ag‐AgCl surface electrodes (with a 10‐mm diameter). EMG signals were amplified and filtered (5–2000 Hz) with a bioamplifier at a sampling rate of 5 kHz. The optimal position of the stimulation coil for the experimental group was determined according to the highest motor evoked potential (MEP). The RMT was determined at the minimum stimulus intensity required to elicit MEP at least 50 mV peak‐to‐peak amplitude above the background EMG activity in at least 5 of 10 consecutive trials in the relaxed APB muscle. In addition, for the control group, the position of the stimulation coil was placed at the left DLPFC in the injured side (position F3 of the 10/20 international electroencephalography system). In the placebo group, the position of the stimulation coil was the same as that in the test group, but the surface of the coil was perpendicular to the 45° angle plane of the scalp tangent of the patient's stimulation target point. Therefore, the patients could hear the sound of magnetic stimulation without the magnetic field being pulsed into the patients' brains.

### 2.4 Evaluation methods

All evaluations included in the entire trial process and were performed by the same rehabilitation therapist from the Rehabilitation Evaluation Department of the Rehabilitation Medicine Department of the First Affiliated Hospital of Jinan University. The rehabilitation therapist has been engaged in PT clinical work for 6 years and was unaware of the grouping of patients and the purpose of the trial during the entire trial. After the patients were enrolled, the GCS and CRS‐R scale assessments, as well as EEG and upper limb SSEP examinations were performed before and after the trial, and then the results of the two assessments were compared and analyzed.

Glasgow Scale (GCS) (Teasdale & Jennett, 1974): The GCS assessment is commonly used clinically to judge the state of consciousness disorder in coma patients. The GCS is composed of the following three aspects: eye opening response, language response and body movement. The highest score is 15 points, indicating clear consciousness; 12–14 points indicate mild disturbance of consciousness; 9–11 points indicate moderate disturbance of consciousness; 8 points or less signifies a coma state. The lower the score, the more severe the disturbance of consciousness.

The coma recovery scale‐revised (CRS‐R) (Giacino et al., 2004): The CRS‐R is a 2004 modified coma recovery scale‐revised (CRS). The CRS‐R consists of 6 subscales, involving auditory, visual, motor, language, communication and arousal levels. We focused on the analysis of the two levels of “hearing and sports,” including 23 hierarchical and orderly scoring standards. The highest score was 23 points, and the higher the score, the lighter the disturbance of consciousness (Schiff, 2012). Regarding the scores, the VS evaluation standard was as follows: CRS‐R score for auditory ≤ 2 points, visual ≤ 1 point, exercise ≤ 2 points, language response ≤ 2 points, communication = 0 points, and arousal ≤ 2 points. MCS evaluation standards were as follows: auditory > 2 points or visual > 1 point or sports > 2 points or verbal response > 2 points or communication > 0 points or arousal > 2 points.

Electroencephalograph (EEG): The digital electroencephalograph imported from Italy was used. Electrodes were placed according to the international 10/20 system, single and bipolar leads were used for tracing, and each tracing was completed within 20 min. The EEG analysis tool of EEGLAB was used to analyze and extract the obtained patient data; mainly, the corresponding amplitude, frequency, waveform and phase data were extracted. The data obtained after analysis were classified into 5 levels according to the Hockaaday (1965) EEG grading standard for disorders of consciousness. The grading standard is as follows: Level I, normal range—the EEG waveform examined is an alpha rhythm or mainly an alpha rhythm, accompanied by a few theta waves; Level II—mild abnormality: the EEG waveform examined is based on theta waves mainly, accompanied by a few delta waves; Level III, moderately abnormal—the EEG waveform examined are delta waves, mixed with theta waves, or mainly alpha waves or delta waves, without other rhythmic activities; Level IV, severe abnormality—the examined EEG waveform consists of diffuse delta waves, accompanied by short‐term electrical resting or some channels scattered in delta waves, and the other channels with electrical resting; Level V, extremely abnormal—the examined EEG waveform consists of nearly all flat waves or no electrical brain activity.

Upper limb somatosensory evoked potentials (SSEPs): The SSEPs use the electromyographic evoked potential meter produced by Oxford, UK, and are acquired by stimulating the median nerve of the wrist with a square wave with a wave width of 200 μs and a frequency of 2 times per second. Evoked potentials were recorded from points of Erb, scalp C4, C3, and neck; the reference electrode was placed at Fpz. The bandpass range was 20–2000 Hz, and the stimulus intensity was 4–10 Ma, which caused slight twitching of the thumb. The analysis time was 50 ms. The CCT (which is the latency between N13 and N20 waves) was measured. The measured evoked potential signals were stored on the computer hard disk, and then the mathematical analysis and processing software MATLAB were used to analyze and process the collected signals. The methods used mainly included coherent average and frequency domain analysis. According to the grading standard of Seel et al. (2010), the SSEP of the tested patients was classified into three grades. The grading standard is as follows: Level I, normal—CCT on both sides of the evoked potential examined is normal; Level II, mild abnormality—the CCT on one or both sides of the evoked potential examined is extended to an abnormal or the CCT on the left and right sides is asymmetric (even if the CCTs on both sides are in the normal range; note that the difference more than 0.8 ms is also called asymmetry); Grade III, severe abnormality—N20 waves on one or both sides of the evoked potential under examination disappeared.

### 2.5 Statistical analysis

Repeated‐measures two‐way ANOVAs were performed to determine the effect of group (test group, control group vs. placebo group) and condition (pretreatment vs. posttreatment) on GCS score, CRS‐R total scores and subscores, EEG reactivity, and SSEPs. Bonferroni post hoc correction was used to check for significant comparisons (significance level α of 0.05). A priori comparisons were made as specified. Normal distribution was tested by the Shapiro–Wilk test (all *p* > .05). The significance level was set at *p* < .05 and group data are presented as mean ± SD in the text and as SEM in the figures.

## 3 RESULTS

### 3.1 Grouping

According to the random number table method, a total of 99 cases were included, with 33 cases in each group. There were no significant differences in the sex, age, injury type, Glasgow Coma Scale (GCS) score, or modified CRS‐R scale (*p* > .05) of the three groups of cases (Table 1).

**TABLE 1:** Demographic and clinical characteristics of all the patients included in the study

Case	Age	Gender	Clinical diagnosis (before)	Clinical diagnosis (after)	Etiology	MRI findings	Months since injury
**Experimental group**							
1	65	F	VS	MCS	Traumatic	Left frontal lobe hemorrhage	3
2	48	M	VS	MCS	Traumatic	Right centroparietal hematoma	3
3	61	M	VS	VS	Traumatic	Left temporal lobe hemorrhage	2
4	76	M	VS	MCS	Traumatic	Left temporal lobe hemorrhage	2
5	49	F	VS	MCS	Traumatic	Left temporal lobe hemorrhage	1
6	55	F	VS	VS	Traumatic	Right thalamic hemorrhage	2
7	78	F	VS	MCS	Traumatic	Right frontal lobe hemorrhage	3
8	46	M	VS	VS	Traumatic	Left temporal lobe hemorrhage	1
9	53	M	VS	VS	Traumatic	Left temporal lobe hemorrhage	1
10	77	F	VS	VS	Traumatic	Left centroparietal hematoma	3
11	37	F	VS	VS	Traumatic	Left frontal lobe hemorrhage	1
1	39	F	VS	Normal	Traumatic	Right centroparietal hematoma	1
13	66	M	VS	VS	Traumatic	Right centroparietal hematoma	2
14	54	M	VS	MCS	Traumatic	Right thalamic hemorrhage	3
15	78	F	VS	Normal	Traumatic	Right thalamic hemorrhage	2
16	48	F	VS	VS	Traumatic	Left temporal lobe hemorrhage	2
17	58	F	VS	MCS	Traumatic	Left temporal lobe hemorrhage	1
18	50	F	VS	MCS	Traumatic	Right thalamic hemorrhage	1
19	48	M	VS	MCS	Traumatic	Right frontal lobe hemorrhage	3
20	45	M	VS	VS	Traumatic	Left temporal lobe hemorrhage	1
21	63	M	VS	VS	Traumatic	Right frontal lobe hemorrhage	3
22	70	F	VS	MCS	Traumatic	Left centroparietal hematoma	2
23	65	M	VS	MCS	Traumatic	Left frontal lobe hemorrhage	3
24	58	M	VS	MCS	Traumatic	Right frontal lobe hemorrhage	2
25	26	M	VS	VS	Traumatic	Left frontal lobe hemorrhage	3
26	68	F	VS	MCS	Traumatic	Right centroparietal hematoma	2
27	49	M	VS	VS	Traumatic	Left frontal lobe hemorrhage	3
28	53	M	VS	VS	Traumatic	Right centroparietal hematoma	2
29	55	F	VS	VS	Traumatic	Left temporal lobe hemorrhage	3
30	38	F	VS	VS	Traumatic	Left frontal lobe hemorrhage	2
31	31	F	VS	VS	Traumatic	Right centroparietal hematoma	1
32	78	F	VS	VS	Traumatic	Left temporal lobe hemorrhage	1
33	69	M	VS	VS	Traumatic	Left temporal lobe hemorrhage	1
**Control group**							
1	51	M	VS	MCS	Traumatic	Left temporal lobe hemorrhage	2
2	51	M	VS	VS	Traumatic	Left frontal lobe hemorrhage	3
3	72	M	VS	VS	Traumatic	Right centroparietal hematoma	3
4	51	M	VS	MCS	Traumatic	Left temporal lobe hemorrhage	2
5	51	M	VS	VS	Traumatic	Left temporal lobe hemorrhage	1
6	59	M	VS	VS	Traumatic	Left temporal lobe hemorrhage	3
7	61	M	VS	MCS	Traumatic	Left frontal lobe hemorrhage	1
8	36	M	VS	VS	Traumatic	Right centroparietal hematoma	2
9	57	F	VS	VS	Traumatic	Left temporal lobe hemorrhage	2
10	24	F	VS	VS	Traumatic	Left temporal lobe hemorrhage	3
11	37	M	VS	VS	Traumatic	Left temporal lobe hemorrhage	3
12	52	M	VS	MCS	Traumatic	Right thalamic hemorrhage	3
13	56	M	VS	VS	Traumatic	Right frontal lobe hemorrhage	2
14	46	M	VS	VS	Traumatic	Left frontal lobe hemorrhage	2
15	19	M	VS	VS	Traumatic	Right centroparietal hematoma	3
16	46	M	VS	VS	Traumatic	Left temporal lobe hemorrhage	1
17	70	M	VS	VS	Traumatic	Left temporal lobe hemorrhage	1
18	56	M	VS	MCS	Traumatic	Left temporal lobe hemorrhage	2
19	30	F	VS	VS	Traumatic	Right thalamic hemorrhage	1
20	46	M	VS	Normal	Traumatic	Right frontal lobe hemorrhage	1
21	76	F	VS	VS	Traumatic	Left temporal lobe hemorrhage	1
22	50	M	VS	MCS	Traumatic	Left frontal lobe hemorrhage	3
23	77	F	VS	VS	Traumatic	Right centroparietal hematoma	2
24	53	M	VS	VS	Traumatic	Left temporal lobe hemorrhage	3
25	38	F	VS	VS	Traumatic	Left temporal lobe hemorrhage	1
26	51	F	VS	MCS	Traumatic	Right centroparietal hematoma	3
27	65	M	VS	MCS	Traumatic	Left temporal lobe hemorrhage	2
28	64	M	VS	VS	Traumatic	Left temporal lobe hemorrhage	2
29	64	M	VS	VS	Traumatic	Left temporal lobe hemorrhage	2
30	42	M	VS	VS	Traumatic	Right thalamic hemorrhage	1
31	78	M	VS	VS	Traumatic	Left frontal lobe hemorrhage	3
32	83	M	VS	VS	Traumatic	Right centroparietal hematoma	2
33	78	M	VS	VS	Traumatic	Left temporal lobe hemorrhage	2
**Placebo group**							
1	53	M	VS	VS	Traumatic	Left temporal lobe hemorrhage	2
2	62	M	VS	VS	Traumatic	Left temporal lobe hemorrhage	2
3	43	M	VS	VS	Traumatic	Right thalamic hemorrhage	3
4	45	F	VS	VS	Traumatic	Right frontal lobe hemorrhage	1
5	77	F	VS	VS	Traumatic	Left temporal lobe hemorrhage	2
6	76	F	VS	VS	Traumatic	Left temporal lobe hemorrhage	3
7	77	M	VS	VS	Traumatic	Right centroparietal hematoma	1
8	69	M	VS	VS	Traumatic	Left temporal lobe hemorrhage	3
9	70	M	VS	VS	Traumatic	Left temporal lobe hemorrhage	3
10	78	F	VS	VS	Traumatic	Left temporal lobe hemorrhage	2
11	52	M	VS	VS	Traumatic	Right thalamic hemorrhage	3
12	51	F	VS	VS	Traumatic	Left temporal lobe hemorrhage	2
13	45	M	VS	VS	Traumatic	Right thalamic hemorrhage	2
14	50	M	VS	VS	Traumatic	Right frontal lobe hemorrhage	3
15	71	M	VS	MCS	Traumatic	Left temporal lobe hemorrhage	1
16	46	M	VS	VS	Traumatic	Left temporal lobe hemorrhage	2
17	39	M	VS	VS	Traumatic	Left centroparietal hematoma	1
18	55	F	VS	VS	Traumatic	Left frontal lobe hemorrhage	1
19	57	M	VS	VS	Traumatic	Right centroparietal hematoma	2
20	59	F	VS	VS	Traumatic	Left temporal lobe hemorrhage	2
21	79	F	VS	MCS	Traumatic	Left temporal lobe hemorrhage	1
22	36	M	VS	VS	Traumatic	Left temporal lobe hemorrhage	1
23	34	M	VS	VS	Traumatic	Right thalamic hemorrhage	3
24	44	F	VS	VS	Traumatic	Right thalamic hemorrhage	3
25	32	M	VS	VS	Traumatic	Right frontal lobe hemorrhage	2
26	79	F	VS	VS	Traumatic	Left temporal lobe hemorrhage	1
27	28	M	VS	VS	Traumatic	Right centroparietal hematoma	1
28	47	F	VS	VS	Traumatic	Left temporal lobe hemorrhage	1
29	66	F	VS	VS	Traumatic	Left temporal lobe hemorrhage	2
30	62	F	VS	VS	Traumatic	Left temporal lobe hemorrhage	3
31	46	M	VS	VS	Traumatic	Right thalamic hemorrhage	2
32	60	F	VS	VS	Traumatic	Left temporal lobe hemorrhage	2
33	41	M	VS	VS	Traumatic	Left temporal lobe hemorrhage	3

### 3.2 GCS score

**FIGURE 1:**
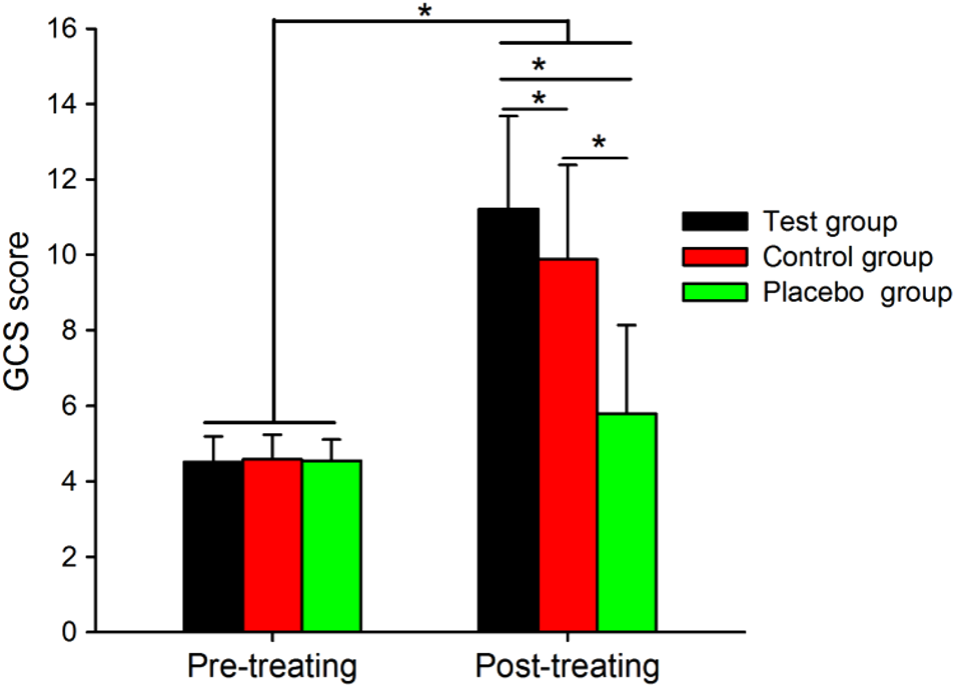
Group data of GCS scores of the test, control and placebo groups in the pretreatment and posttreatment conditions. Error bars indicate the SEs. *p < .05.

Figure 1 shows the results of the GCS score. Note that the GCS score increased after treatment in the test group, control group and placebo group, but the increase in the GCS score was larger in the test group than in the control group and placebo group.

Table 2 shows the results of the GCS scores at pretreatment and posttreatment in the test, control and placebo groups by two‐way repeated‐measures ANOVA. We found that the GCS score was increased to a larger extent after treatment in the test group (*p* < .001) and in the control group (*p* < .001) compared to the increase in the placebo group, and increased in a larger extent in the test group compared to the increase in the control group (*p* = .004). However, there was no change at the pretreatment among the test, control, and placebo groups (all *p* > .05).

**TABLE 2:** GCS scores,control and placebo groups at pretreatment and post treatment conditions(*n* = 33 for each group)

Case	Group	Pretreatment	Posttreatment	*p* Value
GCS scores	Test	4.52 ± 0.67	11.21 ± 2.47	< .01
	Control	4.58 ± 0.66	9.88 ± 2.5	
	Placebo	4.55 ± 0.56	5.79 ± 2.34	

### 3.3 CRS‐R score

**FIGURE 2:**
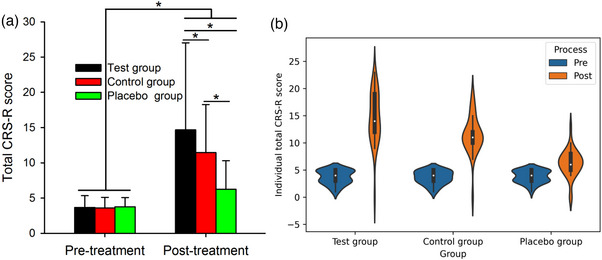
Total CRS‐R scores. (A) Group data (*n* = 33 for each group) showing the value of the total CRS‐R scores of the test, control and placebo groups in the pretreatment and posttreatment conditions. The distributions from individual subjects are also shown by violin plots. (B) Pre represents pretreatment, whereas post indicates posttreatment. Error bars indicate the SEs. *p < .05.

Table 3 shows the results of the CRS‐R total scores and subscores at pretreatment and posttreatment in the test, control, and placebo groups. Figure 2 also illustrates the individual CRS‐R total scores. Note that the CRS‐R total scores and subscores increased after treatment in the test group, control group and placebo group, but the increase in CRS‐R total scores and subscores was larger in the test group than in the control group and in the placebo group.

**TABLE 3:** JFK CRS‐R total scores and subscores of the test, control and placebo groups at pretreatment and posttreatment conditions (*n* = 33 for each group)

Case	Group	Pretreatment	Posttreatment	*p* Value
Auditory function	Test	0.64 ± 0.24	2.91 ± 1.09	< .01
	Control	0.67 ± 0.23	2.52 ± 0.76	
	Placebo	0.7 ± 0.22	1.48 ± 0.45	
Visual function	Test	0.73 ± 0.21	2.67 ± 0.98	< .01
	Control	0.7 ± 0.22	2.09 ± 0.52	
	Placebo	0.76 ± 0.19	1.3 ± 0.34	
Motor function	Test	0.73 ± 0.21	3.48 ± 1.45	< .01
	Control	0.7 ± 0.22	2.64 ± 0.86	
	Placebo	0.76 ± 0.19	1.12 ± 0.74	
Verbal function	Test	0.79 ± 0.17	2.39 ± 0.75	< .01
	Control	0.73 ± 0.21	1.64 ± 0.68	
	Placebo	0.73 ± 0.27	1.24 ± 0.5	
Communication function	Test	0.00 ± 0.00	0.76 ± 0.38	< .01
	Control	0.00 ± 0.00	0.61 ± 0.31	
	Placebo	0.00 ± 0.00	0.15 ± 0.13	
Arousal unction	Test	0.79 ± 0.3	2.45 ± 0.57	< .01
	Control	0.82 ± 0.22	1.97 ± 0.78	
	Placebo	0.82 ± 0.22	0.94 ± 0.5	
Total score	Test	3.67 ± 1.67	14.67 ± 12.35	< .01
	Control	3.61 ± 1.5	11.45 ± 6.82	
	Placebo	3.76 ± 1.31	6.24 ± 4.06	

Post hoc analysis showed that the CRS‐R score of auditory function was larger in the posttreatment condition than in the pretreatment condition in the test group (*p* < .001), control group (*p* < .001), and placebo group (*p* < .001). We found that the CRS‐R score of auditory function was increased to a larger extent after treatment in the test group (*p* < .001) and control group (*p* < .001) compared to the increase in the placebo group, and increased to a larger extent in the test group compared to the increase in the control group (*p* = .024). However, there were no changes at the pretreatment among the test group, control group and placebo group (all *p* > .05).

Post hoc analysis showed that the CRS‐R score of visual function was larger in the posttreatment condition than in the pretreatment condition in the test group (*p* < .001), control group (*p* < .001), and placebo group (*p* = .005). We found that the CRS‐R score of visual function was increased to a larger extent after treatment in the test group (*p* < .001) and control group (*p* < .001) compared to the increase in the placebo group, and increased in a larger extent in the test group compared to the increase in the control group (*p* = .002). However, there were no changes at the pretreatment among the test group, control group and placebo group (all *p* > .05).

Post hoc analysis showed that the CRS‐R score of motor function was larger in the posttreatment condition than in the pretreatment condition in the test group (*p* < .001), and control group (*p* < .001), but not in the placebo group (*p* = .09). We found that the CRS‐R score of motor function was increased to a larger extent after treatment in the test group (*p* < .001) and control group (*p* < .001) compared to the increase in the placebo group, and increased to a larger extent in the test group compared to the increase in the control group (*p* < .001). However, there were no changes at the pretreatment among the test, control, and placebo groups (all *p* > .05).

Post hoc analysis showed that the CRS‐R score of verbal function was larger in the posttreatment condition than in the pretreatment condition in the test group (*p* < .001), control group (*p* < .001), and placebo group (*p* < .001). We found that the CRS‐R score of language function was increased to a larger extent after treatment in the test group (*p* < .001) and control group (*p* = .04) compared to the increase in the placebo group and increased to a larger extent in the test group compared to the increase in the control group (*p* < .001). However, there were no changes at the pretreatment among the test group, control group and placebo group (all *p* > .05).

Post hoc analysis showed that the CRS‐R score of communication function was larger in the posttreatment condition than in the pretreatment condition in the test group (*p* < .001) and control group (*p* < .001) but not in the placebo group (*p* = .11). We found that the CRS‐R score of communication function was increased to a larger extent after treatment in the test group (*p* < .001) and control group (*p* < .001) compared to the placebo group but not in the test group compared to the the increase in the control group (*p* = .23). However, there were no changes at the pretreatment among the test group, control group and placebo group (all *p* > .05).

Post hoc analysis showed that the CRS‐R score of arousal function was larger in the posttreatment condition than in the pretreatment condition in the test group (*p* < .001) and control group (*p* < .001) but not in the placebo group (*p* = .39). We found that the CRS‐R score of arousal function was increased to a larger extent after treatment in the test group (*p* < .001) and control group (*p* < .001) compared to the increase in the placebo group and in the test group compared to the control group (*p* = .007). However, there were no changes at pretreatment among the test group, control group and placebo group (all *p* > .05).

Post hoc analysis showed that the total CRS‐R score was larger in the posttreatment condition than in the pretreatment condition in the test group (*p* < .001), control group (*p* < .001), and placebo group (*p* < .001). We found that the total CRS‐R score was increased to a larger extent after treatment in the test group (*p* < .001) and control group (*p* < .001) compared to the increase in the placebo group and in the test group compared to the increase in the control group (*p* < .001). However, there were no changes at the pretreatment among the test group, control group and placebo group (all *p* > .05).

### 3.4 EEG reactivity

After treatment, the EEG results of the experimental group were one case of brain death, two cases that were the same as before treatment, and the rest were significantly improved.

**FIGURE 3:**
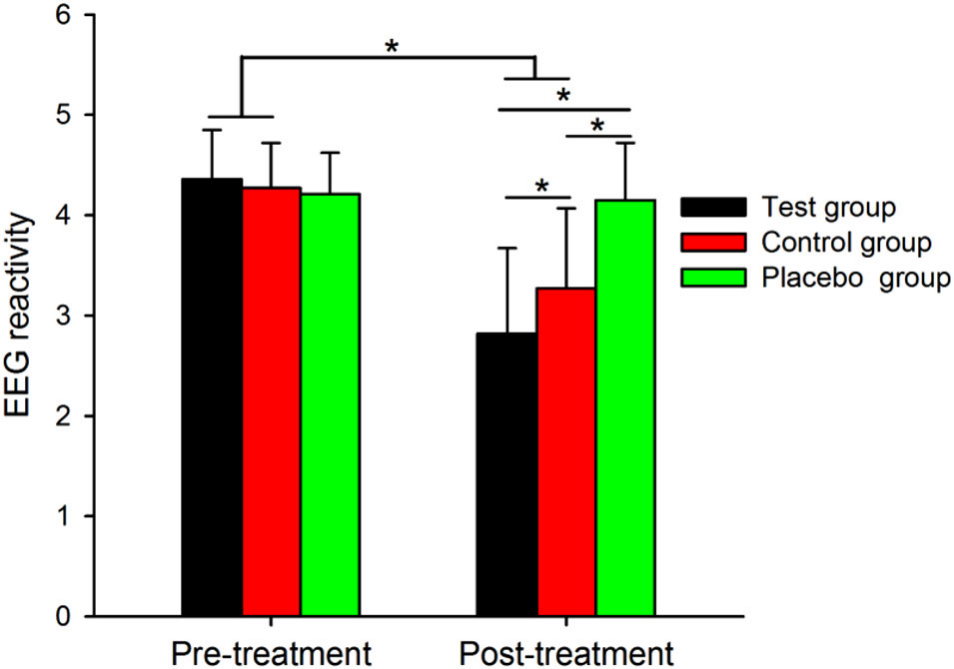
Group data of EEG reactivity of the test, control, and placebo groups in the pretreatment and posttreatment conditions. Error bars indicate the SEs. *p < .05.

Figure 3 shows the EEG reactivity results. Note that EEG reactivity decreased after treatment in the test group, control group and placebo group, but the decrease in EEG reactivity was larger in the test group than in the control group and placebo group.

Table 4 shows the results of the EEG reactivity at pretreatment and posttreatment in the test, control and placebo groups by Two‐way repeated‐measures ANOVA. Post hoc analysis showed that EEG reactivity was smaller in the posttreatment condition than in the pretreatment condition in the test group (*p* < .001) and in the control group (*p* < .001) but not in the placebo group (*p* = .68). We found that EEG reactivity was decreased to a larger extent after treatment in the test group (*p* < .001) and control group (*p* < .001) compared to the decrease in the placebo group and EEG reactivity was decreased to a larger extent in the test group compared to the decrease in the control group (*p* = .002). However, there were no changes at the pretreatment among the test group, control group and placebo group (all *p* > .05).

**TABLE 4:** EEG reactivity, control, and placebo groups at pretreatment and posttreatment conditions (*n* = 33 for each group)

Case	Group	Pretreatment	Posttreatment	*p* Value
EEG reactivity	Test	4.36 ± 0.49	2.82 ± 0.85	< .01
	Control	4.27 ± 0.45	3.27 ± 0.80	
	Placebo	4.21 ± 0.41	4.15 ± 0.57	

### 3.5 SSEP

After treatment, The SSEP index result was 1 case of brain death, 10 cases that were the same as before treatment, and the rest were significantly improved.

**FIGURE 4:**
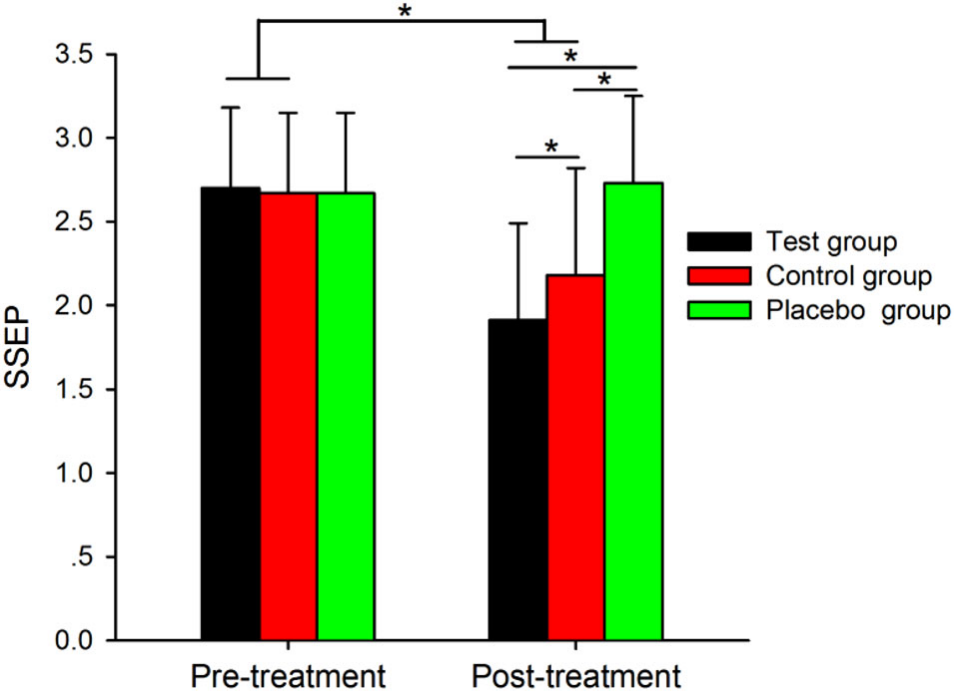
Group data of SSEP of the test, control, and placebo groups at pretreatment and posttreatment conditions. Error bars indicate the SEs. *p < .05.

Figure 4 shows the SSEP results. Note that SSEPs decreased after treatment in the test group, control group and placebo group, but the decrease in SSEPs was larger in the test group than in the control group and placebo group.

Table 5 shows the results of the SEEP at pretreatment and posttreatment in the test, control, and placebo groups by Two‐way repeated‐measures ANOVA. Post hoc analysis showed that SSEPs were smaller in the posttreatment condition than in the pretreatment condition in the test group(*p* < .001) and the control group (*p* < .001) but not in the placebo group (*p* = .59). We found that SSEPs were decreased to a larger extent after treatment in the test group (*p* < .001) and control group (*p* < .001) compared to the decrease in the placebo group and were decreased to a larger extent in the test group compared to the control group (*p* = .036). However, there were no changes at the pretreatment among the test group, control group and placebo group (all *p* > .05).

**TABLE 5:** SEEP, control, and placebo groups at pretreatment and posttreatment conditions (*n* = 33 for each group)

Case	Group	Pretreatment	Posttreatment	*p* Value
SEEP	Test	2.70 ± 0.48	1.91 ± 0.58	<.01
	Control	2.67 ± 0.48	2.18 ± 0.64	
	Placebo	2.67 ± 0.48	2.73 ± 0.52	

## 4 DISCUSSION

In this study, the experimental group underwent 20 Hz rTMS to stimulate the M1 brain area of VS patients. It was found that the clinical awakening effect was more notable than that of the control group and the placebo group. It was mainly manifested in two clinical indicators, namely, the GCS and CRS‐R two clinical scale evaluation indicators. After treatment, the GCS and CRS‐R scores of the experimental group were significantly improved except for one case of brain death that had a score of 0. In addition, two other neuroelectrophysiological indicators were used, namely, EEG and SSEP measures.

The occurrence of consciousness disturbance after head injury is related to ischemia‐hypoxic necrosis of brain tissue. This is because cerebral ischemia and hypoxia will directly cause certain areas of the brain to suspend work—for instance the cerebral cortex is not able to effectively activate—and the brain network foundation related to consciousness is in a state of imbalance. It is generally believed that cognition and awakening are correlate with consciousness. Hinter‐buchner refers to these two as the content of consciousness and the switching system. The content of consciousness refers to the high‐level activities of the cerebral cortex, including behavioral responses such as memory, thinking, orientation, motor, speech, and audiovisual activity. The mechanisms governing consciousness can activate the cerebral cortex, maintain excitement, and keep the body in an awake state. Therefore, effective activation of the cerebral cortex and the regulation of the brain–brain functional network are essential for the arousal of disorders of consciousness. High‐frequency rTMS acts on the cerebral hemisphere of the affected side and directly improves the excitability of the cerebral hemisphere (Emara et al., 2010; Noh et al., 2019). Studies have speculated that the wakefulness‐ promoting effect of high‐frequency rTMS may promote the axon repair of neurons in the affected hemisphere, thereby reactivating neurons in a dormant state or reconnecting brain regions in an isolated state (Bernat, 2006; Tsai et al., 2014). There are also studies that suggest that rTMS can activate or inhibit the activities of cortex‐cortex and cortical‐subcortical neural networks (Lapitska et al., 2009), as well as regulate cortical plasticity (Chen & Udupa, 2009), thereby reshaping perception. For example, Piccione et al. (2011) reported that a patient in a minimum consciousness state for 5 years was given a high‐frequency 20 Hz stimulation frequency, and after 100 rTMS stimulations in 10 sequences, they found that the frequency of patient‐specific meaningful behaviors increased. The EEG output also indicated improvements, and the results suggested that rTMS can improve the attention and response ability of patients with minimal consciousness. In the study by Pape et al. (2009), after 6 weeks of high‐frequency rTMS treatment for patients with severe brain injury in a vegetative state, the brainstem auditory‐evoked V wave latency and the wave difference between the I‐V latency waves were improved. Regarding the selection of the stimulation site, the traditional approach is to stimulate the damaged part of the brain to subsequently stimulate the corresponding functional area in the damaged side, which has a positive effect on awakening. For example, studies have found that rTMS stimulates the prefrontal cortex on the affected side of the brain to help patients recover from a VS to an MCS (Louise‐Bender Pape et al., 2009) or stimulates the M1 area to help patients recover from an MCS‐ to an MCS+ (Piccione et al., 2011).

The M1 area is the high‐level nerve center that controls body movements. It plays a very important role in the planned movements of the body and controls autonomous movements. The M1 area receives and processes peripheral sensory information while outputting the information to the basal ganglia. Nerve information is processed, integrated, and conducted in the cortex‐basal ganglia‐thalamus‐cortex neural circuit, which affects the voluntary and autonomous movement of the body. The execution and adjustment of the system are of great significance. The working principle of rTMS is to use a time‐varying magnetic field to generate an induced magnetic field that induces secondary currents in nearby nerve tissues. The latter activates the cerebral cortex, changes the related physiological processes of the brain tissue, and realizes the positioning of the cerebral cortex function. At the same time, it can also adjust the excitability of local brain tissues, improve local brain hemorheology and cortical metabolism, affect the release and transmission of neurotransmitters in the brain, and promote the repair of damaged brain cells, thereby leading to clinical awakening (Giacino & Trott, 2004). Studies have shown that the cerebral cortex responds differently to different degrees of rTMS at different frequencies. High‐frequency rTMS can excite the cerebral cortex, and low‐frequency rTMS can inhibit it, indicating that the underlying mechanism that mediates the excitability of the cerebral hemisphere may be related to frequency. Therefore, we assume that stimulating the M1 area with high‐frequency rTMS will have a positive effect on the effective activation of the cerebral cortex and the effective regulation of the brain–brain functional network.

Recent research results also support our results. For example, Maas et al. (2008) used 20 Hz rTMS to stimulate the left motor cortex (M1) in a randomized sham‐controlled trial involving seven patients with impaired consciousness and achieved positive results. He believed that stimulating the MI area with high‐frequency rTMS can help the reconstruction of the left parietal cortex, the left inferior temporal cortex and the right DLPFC, thereby improving the brain network foundation of consciousness. Furthermore, Manganotti et al. (2013) and others in a study involving six patients in a VS and those with MCS treated with 20 Hz rTMS found that stimulating the M1 area can induce important neuroelectrophysiological changes in the brain and effectively improve cerebral cortex excitement. In addition, Abelson‐Mitchell (2008) made a case report and found that stimulating the M1 region with high‐frequency rTMS had a positive effect on the recovery of the patient's consciousness and movement. In addition, Li and Jiang (2012) made an active attempt using neuromodulation treatment with high‐frequency rTMS for disorders of consciousness. In their study, 16 patients with disorders of consciousness who were treated with 10 Hz rTMS (left DLPFC) resulted in nine cases of CRS‐R. The scores improved, and it is believed that the increased activity of the parietal and prefrontal neurons may help the recovery of consciousness. However, in another study by Cincotta et al. (2015), there was no improvement of consciousness in UWS/VS patients following 20 Hz rTMS applied at the M1 region. This is mainly because the UWS/VS patients applied in this study are with disorders of consciousness more than 9 months. In our study, the patients are with 1–3 months.

In addition, there are some opinions that rTMS changes cortical metabolism and cerebral blood flow by changing the excitability of the local cerebral cortex, affecting neurotransmitters and their transmission in the brain, increasing the reversibility of damaged cells, and promoting the recovery of brain function (Potapov et al., 2016; Sveen et al., 2022). It has been reported in the literature that after rTMS stimulates the right prefrontal lobe of patients with depression, the local cerebral blood flow increases (Iaccarino et al., 2015); for healthy volunteers, there are significant changes in SSEPs and local cerebral blood flow after single‐phase rTMS stimulation (Knight et al., 2005). There are also research opinions that the early rTMS technology is mainly used to observe the motor evoked potentials of patients with impaired consciousness (Potapov et al., 2016). A number of studies have proven that rTMS is helpful in evaluating the recovery of potential motor functions of patients with impaired consciousness, but it is used in evaluating the recovery of consciousness There is still a lack of effective evidence for the degree and prognosis (Kondziella et al., 2020; Manganotti et al., 2013). Other studies (Giacino & Whyte, 2005; Lombardi et al., 2002; Seel et al., 2010) believe that rTMS exerts a deeper impact on the cerebral cortex, such as increasing the release of dopaminergic substances and regulating the synchronous oscillations of the brain, thereby promoting consciousness.

In summary, rTMS stimulates the M1 area and is effective for improving the clinical consciousness of patients with impaired consciousness. The possible mechanism includes changes in cerebral blood flow speed and neuroelectrophysiological changes by stimulating the cerebral cortex, thereby improving the activity of brain cells. Therefore, the regulation of nerve excitement, the promotion of dopamine secretion, and the regulation of brain wave resonance promote the recovery of brain injury functions and helps in the recovery of consciousness.

## AUTHOR CONTRIBUTIONS

LS, YH, ZC, and JL conceived and designed the study. LS, YH, and YL acquired the data. LS, YH, YL, ZC, and JL analyzed and interpreted the data. All authors contributed to the writing of the manuscript, read, and approved the final version.

## CONFLICT OF INTEREST STATEMENT

The authors declare no competing interests.

### PEER REVIEW

The peer review history for this article is available at https://publons.com/publon/10.1002/brb3.2971.

## Data Availability

All data discussed in the current work have been provided. Additional data not provided can be made available by the authors upon request.
